# Towards a mechanistic understanding of reciprocal drug–microbiome interactions

**DOI:** 10.15252/msb.202010116

**Published:** 2021-03-18

**Authors:** Michael Zimmermann, Kiran Raosaheb Patil, Athanasios Typas, Lisa Maier

**Affiliations:** ^1^ Structural and Computational Biology Unit European Molecular Biology Laboratory Heidelberg Germany; ^2^ The Medical Research Council Toxicology Unit University of Cambridge Cambridge UK; ^3^ Genome Biology Unit European Molecular Biology Laboratory Heidelberg Germany; ^4^ Interfaculty Institute of Microbiology and Infection Medicine University of Tübingen Tübingen Germany; ^5^ Cluster of Excellence ‘Controlling Microbes to Fight Infections’ University of Tübingen Tübingen Germany

**Keywords:** antibiotics, antimicrobials, human gut microbiome, metagenomics, microbial community, Microbiology, Virology & Host Pathogen Interaction

## Abstract

Broad‐spectrum antibiotics target multiple gram‐positive and gram‐negative bacteria, and can collaterally damage the gut microbiota. Yet, our knowledge of the extent of damage, the antibiotic activity spectra, and the resistance mechanisms of gut microbes is sparse. This limits our ability to mitigate microbiome‐facilitated spread of antibiotic resistance. In addition to antibiotics, non‐antibiotic drugs affect the human microbiome, as shown by metagenomics as well as *in vitro* studies. Microbiome–drug interactions are bidirectional, as microbes can also modulate drugs. Chemical modifications of antibiotics mostly function as antimicrobial resistance mechanisms, while metabolism of non‐antibiotics can also change the drugs’ pharmacodynamic, pharmacokinetic, and toxic properties. Recent studies have started to unravel the extensive capacity of gut microbes to metabolize drugs, the mechanisms, and the relevance of such events for drug treatment. These findings raise the question whether and to which degree these reciprocal drug–microbiome interactions will differ across individuals, and how to take them into account in drug discovery and precision medicine. This review describes recent developments in the field and discusses future study areas that will benefit from systems biology approaches to better understand the mechanistic role of the human gut microbiota in drug actions.

## Graphical abstract

Bidirectional interactions have been reported between drugs and the gut microbiome. This Review discusses recent developments in the field as well as future study areas to better understand the mechanistic role of the human gut microbiota in drug actions.

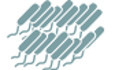

## Introduction

**Figure 1:**
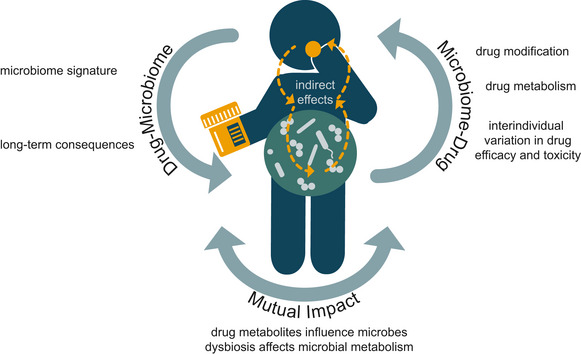
Overview on the drug–microbiome–host triad and their interactions Left: The intake of drugs can have a direct influence on individual members of the gut microbiome (classic example: antibiotics) but can also change the composition and functionality of the microbiome through indirect, host‐mediated ways (example: proton‐pump inhibitors, which might alter the microbiome composition by increasing the gastric pH). Right: Intestinal bacteria can modify and metabolise drugs. In addition, the microbiome can indirectly modulate host xenobiotic metabolism in the liver. Furthermore, there is crosstalk between all these interactions. Ultimately, these complex interactions can possibly have negative health consequences and cause interpersonal differences in treatment outcomes.

**Figure 2:**
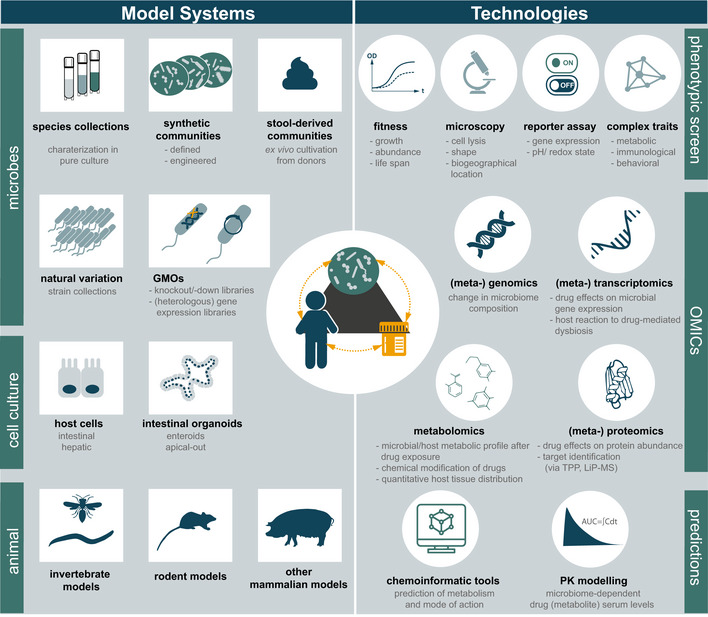
Systems approaches to study drug–microbiome–host interactions Left: A wide variety of model systems can be used to study drug–microbiome–host interactions. On the microbial side, (possibly genetically modified) isolates in pure culture or synthetic or stool‐derived microbial communities are applied. On the host side, simple cell culture systems, intestinal organoids but also different animal models can be employed. Right: Diverse technologies help to decipher drug–microbiome–host interactions. Approaches can be broadly divided into phenotypic characterization, OMICs approaches, and model‐based predictions. Depending on the research question, appropriate model systems and suitable technologies can be combined. TPP: thermal proteome profiling, LiP‐MS: limited proteolysis‐coupled mass spectrometry.

Our understanding of how the human gut microbiota contributes to health and disease, and how it changes over time, life stages, different geographic regions, and in response to environmental factors has increased dramatically over the last decade (The Integrative HMP (iHMP) Research Network Consortium, 2019; Pasolli *et al*, 2019; Nayfach *et al*, 2019; Falony *et al*, 2016). The current consensus is that the gut microbiome has a highly individualized composition, especially at the bacterial strain level (Franzosa *et al*, 2015). Further, healthy individuals retain a largely stable microbiota composition for most of their adulthood (Sommer *et al*, 2017; Mehta *et al*, 2018). This composition is established in early stages of life (Bäckhed *et al*, 2015; Wampach *et al*, 2017) and is dependent more on the environment than on host genetics (Rothschild *et al*, 2018). Hence strong perturbations, such as dietary shifts and antibiotic consumption, can unbalance microbiome stability, with so far unpredictable recovery (Willing *et al*, 2011; Falony *et al*, 2016; Lynn *et al*, 2018). On the other hand, chemical modification of therapeutic compounds by intestinal bacteria can influence the therapeutic effect of drugs (Fig 1). We have only recently begun to explore these complex, bidirectional interactions between our resident microbes and medication. In this review, we provide an overview of the different systems‐level approaches that can be employed to gain insights into the drug–microbiome–host triad (Fig 2). A better and more systematic understanding of these interactions and their underlying molecular constituents can be instrumental for diagnostic, prognostic, and ultimately, therapeutic applications.

## Therapeutic drugs alter the gut microbiome composition

### Evidence from metagenomic‐based cohorts and clinical studies—the top‐down approach

Exploring the factors that explain inter‐individual differences in the intestinal microbiome composition across large population cohorts have repeatedly identified medication as a main contributor (Falony *et al*, 2016; Ticinesi *et al*, 2017; Jackson *et al*, 2018; Vich Vila *et al*, 2020). Although such studies have been insightful and have revealed the cumulative and dramatic impact medication has on the gut microbiome composition, they are still underpowered for separating the effects of individual drug classes. To begin stratifying these effects, one can broadly separate drugs to antimicrobials, developed to target microbes, and to drugs designed to interact with human/host targets, here referred to as human‐targeted drugs.

Antimicrobial drugs comprise antibiotics, antifungals, antiprotozoals, antivirals, and anti‐archaeals. These compounds target proteins that are typically absent in the host or are clearly distinguishable from their human homologues, yet they are often present in commensal microbes colonizing the human body. As a consequence, antimicrobials can “collaterally damage” the microbiome and thereby have mild to severe side effects to patients (Kuhn *et al*, 2016). This has been best studied for antibiotics, with clinical and animal studies illustrating changes in the gut microbiome composition and physiological host parameters, such as metabolic, cognitive, and immune functions (Cho *et al*, 2012; Cox *et al*, 2014; Hwang *et al*, 2015; Fröhlich *et al*, 2016; Hagan *et al*, 2019). Initial data indicate that the microbiota of healthy patients can partially rebound post‐antibiotic treatment (Rashid *et al*, 2015; Palleja *et al*, 2018). However, it remains unclear whether this is true for a broader and/or more diverse population, and what are the links to antibiotic classes, initial microbiome composition and treatment duration. Similarly, our knowledge on the target spectra, mode of action, and resistance mechanisms of the different classes of antibiotics and their specific effect on gut commensal bacterial species is scarce (preprint: Maier *et al*, 2020). To gain mechanistic insights into these matters, assays, tools, and test systems from decades of antibiotic research on pathogens can be capitalized and adapted to study gut commensal species in pure culture, within microbial communities and within the host, especially at a systematic level (Fig 2) (Maier & Typas, 2017). Such detailed mechanistic knowledge can help design better and more precise strategies to prevent or revert antibiotics‐caused "collateral damage," which at the moment are based on generic processes with limited success and/or adverse outcomes, such as fecal transplantation or administration of probiotics (Zmora *et al*, 2018; Suez *et al*, 2018; DeFilipp *et al*, 2019) (Box 2).

For host‐targeted drugs, increasing evidence suggests that they are associated with shifts in gut microbiome composition. Known examples span a broad range of therapeutic classes and include the antidiabetic metformin, proton‐pump inhibitors, antipsychotics, non‐steroidal anti‐inflammatory drugs, paracetamol, opioids, selective serotonin reuptake inhibitors, laxatives, and statins (Le Bastard *et al*, 2018; Jackson *et al*, 2018; Kummen *et al*, 2020; MetaCardis Consortium *et al*, 2020). These shifts are not necessarily unfavorable for the host. In certain cases, host‐targeted drugs can diversify the gut microbiome (MetaCardis Consortium *et al*, 2020)—a feature generally linked to a healthy microbiome. However, the functional implications of these taxonomic shifts, for example in terms of altered metabolic capacities and/or antibiotic resistance repertoires, need to be assessed separately for each compound (Vich Vila *et al*, 2020).

Current clinical studies of the effects of medication on the gut microbiome have mostly been cross‐sectional, while interventional or longitudinal approaches and comparisons to treatment‐naïve but diseased control groups are often missing. As a result, it is difficult to differentiate between disease‐mediated and drug‐related effects. This issue is exemplified by the antidiabetic drug metformin. The drug shows limited oral bioavailability, resulting in high intestinal drug concentration. It was one of the first non‐antibiotic drugs that was shown to influence gut microbiome composition (Napolitano *et al*, 2014) and revealed the need to stratify for treatment when interpreting microbiome signatures (Forslund *et al*, 2015). At the same time, this finding stimulated causal studies that directly linked compositional shifts to the improvement of metabolic dysfunction and hyperglycemia (Wu *et al*, 2017). One proposed mechanism involves metformin decreasing the relative abundance of *Bacteroides fragilis* and downregulating its associated bile salt hydrolase activity. This leads to an accumulation of glycoursodeoxycholic acid, which inhibits the intestinal farnesoid X receptor (FXT) signaling and thereby improves various metabolic outcomes in mice, including hyperglycemia (Sun *et al*, 2018). Other proposed mechanisms to explain the microbiome‐mediated hypoglycemic effect of metformin include the microbial production of short‐chain fatty acids, promotion of gut barrier integrity and increased secretion of gut hormones such as glucagon‐like peptide 1 and peptide YY (PYY) (reviewed in Pryor *et al*, 2020). Remarkably, several model systems such as *Caenorhabditis elegans* (Cabreiro *et al*, 2013), mice (Shin *et al*, 2014), and rats (Bauer *et al*, 2018) were instrumental in elucidating these metformin–microbiome–host interactions, highlighting the translation of these phenomena between evolutionarily distant organisms and demonstrating the utility of different model organisms to study these interactions. In contrast to metformin, we are far from dissecting the interaction of the vast majority of host‐targeted drugs with gut microbes. It remains unclear whether these drugs act directly on the microbes, what is their spectrum and underlying molecular interactions, and what is the impact on the microbiome as a whole, on the drug’s therapeutic action and on the host. To close this knowledge gap and optimize drug therapies, further well‐designed clinical studies are needed, which must be seamlessly coordinated with bottom‐up approaches (Fig 2).

### Ex vivo studies—accelerating mechanistic understanding of drug–microbiome interactions by reducing the complexity and increasing the throughput—the bottom‐up approach

While clinical studies provide an excellent global picture of drug effects on the microbiome, *ex vivo* approaches allow for a systematic, controlled, and question‐specific dissection of these interactions at various scales ranging from molecules to inter‐organismal interactions. Recent advances in high‐throughput approaches for the cultivation of fastidious anaerobes (Box 1) allowed the first systematic studies of the effects of drugs on intestinal microbes. A large‐scale *in vitro* screen of 1,200 marketed drugs showed direct impact on the growth of at least one of forty tested human gut commensal species for 78% of the antibacterial drugs, 53% of other antimicrobials, and 24% of the human‐targeted drugs (Maier *et al*, 2018). Although drugs across all therapeutic classes had a direct impact on gut commensal species, the effect was most pronounced for antimetabolites, antipsychotics, and calcium‐channel blockers. Some of these compounds, such as antimetabolites, target conserved enzymes and pathways in prokaryotes and eukaryotes and thus, likely have the same mode of action in gut commensals as in host cells. However, for the vast majority of human‐targeted drugs with activity against gut bacteria, their bacterial targets remain obscure. Identifying microbial targets for these drugs will open new possibilities for repurposing them as antibacterials and/or for mitigating their collateral damage on gut bacteria. Intriguingly, human‐targeted drugs impacting microbes *in vitro* resembled antibiotics with respect to their reported side effects in clinics, providing initial evidence that they also impact gut commensals *in vivo*. Moreover, antibiotic‐resistant microbes were in general also more resistant to human‐targeted drugs, suggesting that resistance mechanisms against antibiotics and non‐antibiotics at least partially overlap. Initial profiling of these common resistance mechanisms revealed efflux pumps, transporters and detoxifications mechanisms. Other activities, such as cell envelope properties, stress responses and target modification are also likely involved. Precisely mapping this level of cross‐resistance and collateral sensitivity (*i.e*., resistance to one drug providing sensitivity to another) is vital to mitigate the risks human‐targeted drugs may entail for antibiotic resistance and to exploit collateral sensitivity opportunities to delay, prevent or revert antibiotic resistance (Pál *et al*, 2015; Baym *et al*, 2016). To this end, a number of established systems approaches can be specifically geared to deconvolute drug targets and reveal resistance mechanism, as demonstrated for chemical genetics (Cacace *et al*, 2017; Kintses *et al*, 2019), proteomics (thermal proteome profiling (Mateus *et al*, 2020), limited proteolysis‐coupled mass spectrometry (Schopper *et al*, 2017), and metabolomics (Zampieri *et al*, 2018) (Fig 2).

Box 1 **Representative microbes and microbiomes**

**A: Representative microbes**

The significance of systemic mapping of drug–microbiome interactions increases with the number of representative microbes tested. Consequently, comprehensive species and strain collections are essential. The benefit of such collections further increases, the better the isolates are characterized (*e.g*., genome sequence), and the more detailed metadata information is provided (*e.g*., health status of the host).

***Gut microbiome isolate collections***

The compilation of such collections usually follows certain selection criteria—such as being representative for the gut microbiome of healthy individuals—and focuses on type strains, which are obtained from publicly available strain collections such as DSMZ, ATCC/BEI Resources, *etc*. (www.dsmz.de, http://www.atcc.org, www.beiresources.org) (e.g., Tramontano *et al*, 2018). Further collections are needed that are representative for other body sites, certain diseases, age‐groups, ethnicities, food preferences, etc.. While most concentrate on maximizing phylogenetic diversity of prevalent and abundant species, for a global picture it is also important to capture rare species and species diversity (*i.e*., strain‐level variation).

***Strain‐level variation***

Current studies only phenotype one or few strains per species, usually starting with type strains. For most tested species, it is unknown how representative they are. Although pangenomes can be estimated for many gut species (Zou *et al*, 2019), it is unclear how this translates into phenotypic variation. However, previous work suggests that drug metabolism and drug sensitivity are strain‐specific traits (Koppel *et al*, 2018; preprint: Maier *et al*, 2020) and that functional strain differences can impact human health. Such observations underline the importance of sampling many strains per bacterial species. Several efforts have been recently made toward this aim by collecting hundreds of human gut bacterial isolates. In the future, such collections need to continue expanding to cover strain and species diversity—for example, many unknown species are predicted from metagenome‐assembled genomes (Almeida *et al*, 2019; Pasolli *et al*, 2019; Nayfach *et al*, 2019).

Recent examples for such libraries include:

1. Broad Institute‐OpenBiome Microbiome Library (Poyet *et al*, 2019).
2. Culturable Genome Reference (CGR) Collection (Zou *et al*, 2019).
3. Human Gastrointestinal Bacteria Culture Collection (HBC) (Forster *et al*, 2019).
4. Global Microbiome Conservancy (http://microbiomeconservancy.org).

***Collection of coexisting isolates from the same host***

Instead of collecting and phenotyping strains from a large number of different individuals, strain collections can originate from a single person (Goodman *et al*, 2011; Coyne *et al*, 2014). As these co‐resident strains are collected from the same human host, they capture the co‐evolved and coexisting strain‐level diversity within one individual. Personalized collections are of particular value for investigations of inter‐individual differences in drug–microbiome interactions.

**B: Microbiomes**

The number of different community compositions to be examined scales almost infinitely. To tackle this challenge, two fundamentally different approaches can be pursued: synthetic communities can be assembled starting from axenic bacterial cultures (bottom‐up approach) or natural, self‐assembled communities, *e.g*., derived from human stool can be utilized (top‐down approach).

***Synthetic communities***

Reductionist consortia of defined organisms are assembled in modular ways, either donor‐specific or pooled. Individual community members are usually well‐characterized and ideally genetically tractable. Systematic manipulations of the strain and genetic composition of synthetic communities enable the identification of causal links between the composition and observed community phenotypes (Shetty *et al*, 2019).

***Stoolbanks***

Stool samples provide a non‐invasive starting point for studying the complex, self‐assembled human microbiome (Bolan *et al*, 2016) and can be incubated with drugs *ex vivo* (Maurice *et al*, 2013; van de Steeg *et al*, 2018). Recently, so‐called “stoolbanks” became more sophisticated in order to promote accessibility to fecal microbiota transplantation in clinical practice (Cammarota *et al*, 2019). But they can also be used for research purposes, especially if they are open‐access and non‐profit, such as OpenBiome. Subsequent microbiome preservation efforts aim for long‐term storage: for example, the “The Microbiota Vault” (www.microbiotavault.org) is a project to conserve the microbial diversity associated with our bodies and environments for future generations.

In both setups, key functional and compositional profiles of the gut microbiota need to be maintained, for example in continuous flow bioreactor systems or microfluidic gut models (Guzman‐Rodriguez *et al*, 2018). As these technically laborious systems are challenging to adapt to high‐throughput workflows, continuous dilution batch cultures in multi‐well formats have been successfully applied to screen drug effects on microbial communities (Venturelli *et al*, 2018; Li *et al*, 2019).

**Figure 3:**
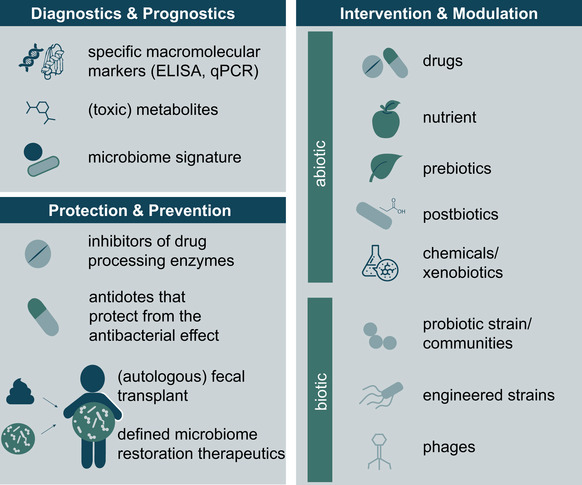
Applications of knowledge gain from studying drug–microbiome–host interactions Diagnostics and Prognostics: Microbiome‐derived biomarkers (macromolecules, metabolites and compositions) can be used to diagnose diseases, but also for prognosis of the disease course or to predict treatment success. Protection and Prevention: Various measures can be applied to reduce undesired drug effects on the microbiome or to suppress chemical drug modifications by intestinal bacteria. With better understanding of the drug–microbiome–host triad, interventions of increased specificity can be employed (i.e., from fecal transplants to defined restoration therapeutics). Intervention and Modulation: There are both abiotic and biotic approaches to influence the microbiome, its functional output and consequently drug–microbiome–host interactions. For more detailed explanations, see Box 2.

The numerous interactions observed between human‐targeted drugs and gut microbes *in vitro* beg the question of whether they are relevant *in vivo*. For example, it is unclear whether microbes alone similarly respond to drugs as when part of a community, and how the spatially structured intestinal environments and drug concentration gradients inside the host affect drug response. One way to leverage drug–microbiome interactions to the community level is to test assembled (“synthetic”) communities (Box 1). Microbes can behave the same in communities as in an axenic culture (the drug being as effective against them) or can have communal emergent properties: be more protected (cross‐protection) or sensitized (cross‐sensitization) to the drug. It is currently unclear how often such emerging communal properties occur and/or what drives them. Drug chemical modification can lead to both cross‐protection (Vega & Gore, 2014) and cross‐sensitization (Roemhild *et al*, 2020), but also other less direct effects could elicit similar results: the change in physiological stage of the bacterial cells (*e.g*., stress responses and transporters induced at the community level), changes of environment (*i.e*., pH changes (Ratzke & Gore, 2018)), or the opening of niches in a competitive environment. To investigate such responses systematically, robust high‐throughput ways are needed to grow communities (Box 1) and to follow species abundance, ideally at an absolute quantification level (*e.g*., by metaproteomics (Li *et al*, 2020), Fig 2). Understanding the frequency and molecular drivers of such interactions will be of paramount importance to exploit or mitigate microbiome‐mediated drug effects in clinics (Fig 3).

## Microbiome effects on drugs

### Microbes alter the chemistry of drugs and drug metabolites

Given the structural similarity between small molecule drugs and endogenous metabolites, the fact that many drugs are derived from natural products, and the large enzymatic potential of the microbiome, microbial drug metabolism is to be expected. Indeed, already in the early 20^th^ century the drug prontosil was found to require bacterial conversion to unfold its antibiotic effects (Fuller, 1937). Since then, accumulating evidence suggests that microbial modification of drugs and drug metabolites seems to be the rule rather than the exception. Such microbial drug metabolism can result in the same or different chemical products as the human metabolic enzymes, leading to drug activation (*e.g*., sulfasalazine, Sousa *et al*, 2014), inactivation (*e.g*., L‐dopa and digoxin (Lindenbaum *et al*, 1981; Haiser *et al*, 2013; Maini Rekdal *et al*, 2019)) or toxicity (*e.g*., sorivudine and brivudine, (Zimmermann *et al*, 2019a; Nakayama *et al*, 1997). In addition to drug molecules, drug metabolites are also subject to microbial metabolism. Phase II drug metabolites (produced by conjugation reactions) have been found to be deconjugated to their precursor molecules (*i.e*., phase I metabolites (Wallace *et al*, 2010) or original drug molecules (Taylor *et al*, 2019)) by microbes. More importantly, these types of microbial metabolism can impact pharmacokinetics, in particular the intestinal abundance of drug and drug metabolites, and thereby alter drug response and toxicity (Wallace *et al*, 2010; Taylor *et al*, 2019). Since differences in microbiome‐encoded genetic contents far exceed genetic differences between human individuals, it is very likely that the microbiota composition may be behind a large fraction of person‐to‐person variation in drug response, especially in terms of drug side effects. In the following paragraphs, we will discuss various approaches to investigate microbiome drug metabolism, its impact on drug response and potential avenues to harness microbiome drug metabolism to improve therapeutic drug interventions. The latter would undoubtedly present an opportunity for the pharmaceutical industry and precision medicine applications in clinics.

### Systematic studies reveal extensive microbial drug metabolism

A compound’s metabolism in the human body is a decisive factor for its success during preclinical and clinical drug development. To assess drug metabolism early in drug discovery pipelines, numerous *in vitro* and *in silico* protocols have been developed and standardized. New technologies, such as microfluidics screens and machine learning predictions have been recently incorporated in such pipelines (Kirchmair *et al*, 2015; Eribol *et al*, 2016). The use of cellular or cell‐free enzyme preparation (*e.g*., cytosolic and microsome isolations) enables systematic *ex vivo* high‐throughput screens for the metabolism of hundreds of compounds in parallel (Williamson *et al*, 2017; Underhill & Khetani, 2018). The results of such systematic assays, together with insights from *in vivo* drug metabolism, are the basis for rule‐based and machine learning computational methods to predict xenobiotic metabolism (Djoumbou‐Feunang *et al*, 2019; de Bruyn Kops *et al*, 2019).

In contrast to human drug metabolism, comparable large‐scale data sets for microbiome drug metabolism are mostly lacking, limiting the information available to build predictive models of microbial drug modifications. To circumvent this limitation, several research groups have used information on primary and secondary metabolism to infer potential drug modification reactions based on biochemical reactions and substrate structures (Klünemann *et al*, 2014; Guthrie *et al*, 2019). Although this approach is consistent with the chemical similarity between drugs and endogenous compounds, it suffers from the fact that the genes, biochemistry, and lifestyle of most gut microbiome members are poorly characterized (Almeida *et al*, 2019). This makes it also challenging to define a (standardized) set of microbiome‐derived species/strains/enzymes to test their activity against drug molecules, as it exists for human drug‐metabolizing enzymes. As a workaround, two recent studies have cultured complete human fecal communities to test their drug‐metabolizing capacity *ex vivo* with a panel of up to 438 different compounds (van de Steeg *et al*, 2018; Javdan *et al*, 2020). This experimental setup has the advantage that microbial community members do not have to be selected *a priori* and encompasses microbial interactions that can impact drug metabolism, as shown for sequential L‐dopa metabolism by two different species (Maini Rekdal *et al*, 2019). A challenge of this approach is the uneven strain distribution in isolated microbial communities, which may mask and underestimate the metabolic potential of microbes found at low abundance *ex vivo*, but may very well be active and relevant *in vivo*. Comparable to the described systematic bottom‐up approach to test drug activity on representative panels of bacteria in isolation (Maier *et al*, 2018), similar efforts have been employed to deduce their metabolic activity against a large panel of drugs (Zimmermann *et al*, 2019b). Testing microbial communities or single bacterial strains, up to 65% of the assayed drugs were metabolized, suggesting that the microbial drug metabolism is a far more common phenomenon than the few anecdotal examples collected over the last few decades (reviewed in Wilson & Nicholson, 2017).

### Gaining molecular insights into microbial drug metabolism

*Ex vivo* drug transformation assays with fecal communities isolated from different individuals have demonstrated vast interpersonal differences in the communities’ drug‐metabolizing capacity (Zimmermann *et al*, 2019b) (Fig 2), which are corroborated by differences in the drug‐metabolizing potential for different bacterial species and strains (Lindenbaum *et al*, 1981; Haiser *et al*, 2013; Zimmermann *et al*, 2019b). These findings suggest that the molecular mechanisms of microbial drug transformation need to be identified to predict the drug‐metabolizing capacity of an individual's microbiome. To identify microbial enzymes and pathways responsible for drug conversion, several systems approaches have been applied. Based on the assumption that metabolic pathways are often transcriptionally induced by their substrates, transcriptional comparison in the presence and absence of a given drug can be performed. This approach was successfully applied to identify the enzymes of *Eggerthella lenta* (DSM 2243) and *Escherichia coli* (K12) that metabolize digoxin (Haiser *et al*, 2013) and 5‐fluoruracil (preprint: Spanogiannopoulos *et al*, 2019), respectively. Gain‐of‐function and loss‐of‐function genetic screens have been combined with mass spectrometry‐based analytics to systematically identify genes involved in microbial drug metabolism (Zimmermann *et al*, 2019a, 2019b) (Fig 2). Drug‐specific chemical probes have also been employed to probe enzyme activity and to pull down enzymes conveying a drug conversion of interest, as elegantly applied for the identification of beta‐glucuronidases (Jariwala *et al*, 2020). Finally, computational approaches based on metabolic reaction networks, comparative genomics of bacterial isolates, or microbiome composition have been employed to identify possible genetic factors responsible for drug metabolism (Klünemann *et al*, 2014; Mallory *et al*, 2018; Guthrie *et al*, 2019). Once identified, microbial genes involved in drug metabolism can serve as potential biomarkers to quantitatively predict the drug metabolic capacity of a given microbial community (Zimmermann *et al*, 2019b) (Fig 3), opening new paths for understanding the impact of microbial drug metabolism on the host and eventually its role in the interpersonal variability in drug response.

## The role of the host

Interactions between drugs and microbes identified *in vitro* need to be validated in the host context, to establish that microbes and drug meet at relevant concentrations and at the same location. Additional interactions that are usually not adequately reflected by *in vitro* systems but are relevant in the host context include dietary interactions, host drug metabolism, immune responses, and the presence of endogenous host molecules. Trying to understand the molecular mechanisms that govern the mutual interactions between microbiome and host and trying to explain the compositional adaptations of the microbial community and altered physiology of the host is at the very heart of microbiome research. Which environmental and host factors shape the composition and the functional output of the microbiome? How do altered microbiome composition and functions affect the host? Altogether, the consequences of microbiome–drug–host interactions need to be understood at a molecular level in order to allow harnessing them and applying them to improve therapy (Fig 3). Below, we discuss suitable approaches for studying microbiome–drug–host interactions (Fig 2).

### In vitro approaches

Microbial communities can interact with and affect the host with peptides/proteins (Gil‐Cruz *et al*, 2019), RNA (Liu *et al*, 2016), and metabolites (Uchimura *et al*, 2018; Koh & Bäckhed, 2020). In the context of microbiome–drug–host interactions, in particular in the case of small molecule drugs, metabolite‐based interactions seem natural. Decades of pharmacological research have led to the development of *in vitro* approaches to systematically screen for molecules with a potential effect on the host. Some of which have also been successfully applied to study metabolic microbiome–host interactions. Membrane‐bound G‐protein‐coupled receptors (GPCR) are a prime target for pharmacological interventions, currently representing more than one‐third of the targets for prescribed drugs (Rask‐Andersen *et al*, 2011). These molecular sensors are omni‐present in mammalian hosts, bind ligands from their environment, and transduce the signal through molecular cascades to change cell physiology. Several studies have recently been published employing high‐throughput GPCR activation assays to screen for microbiome‐produced GPCR ligands (Cohen *et al*, 2017; Colosimo *et al*, 2019; Chen *et al*, 2019). Each of these studies started with metabolites extracted from microbial cultures, which were then tested on engineered GPCR‐reporter cell lines to pinpoint receptor activation. Strikingly, these studies identified microbiome‐derived ligands for yet uncharacterized, so‐called orphan GPCR, which are of particular interest to potentially expand the drug target space. Following the same principle, reporter cell lines for the activation of nuclear receptors, another major target class of drug targets, have been employed to identify microbiome‐derived ligands of human receptors (Estrela *et al*, 2019). These studies illustrate the applicability and power of systematic screens based on human cell lines, initially developed in drug discovery pipelines, to map the chemical interactome between the microbiome and the host. Following these examples, similar screening approaches could be applied to the analysis of different receptor classes, metabolic activity, and transporter specificity. Clear strengths of these assays include their reductionist character, mechanistic insights, and high‐throughput capacity, whereas the lack of tissue context and physiological relevance represent obvious limitations.

Intestinal enteroids and organoids overcome this limitation through differentiation of stem cells into specific intestinal cell types, such as enterocytes and goblet cells, forming crypt macrostructures, and encompass intestinal properties, such as barrier functions (Sato *et al*, 2009; Yin *et al*, 2014; Pearce *et al*, 2018). Such organ culture systems have been used to study the interactions of human enteric tissue with pathogenic and commensal bacteria (Lukovac *et al*, 2014; Pleguezuelos‐Manzano *et al*, 2020). Furthermore, these systems have been successfully employed to study the effect of bacterial surface or secreted molecules, such as lipopolysaccharides and muramyl‐dipeptides (Nigro *et al*, 2014; Naito *et al*, 2017), and of microbiome‐derived small molecules, such as short‐chain fatty acids and indolacrylic acid (Park *et al*, 2016; Wlodarska *et al*, 2017; Pleguezuelos‐Manzano *et al*, 2020). Paired with microfluidic technology, assays co‐culturing microbes with host tissues have been developed to create artificial gut systems on a chip, enabling systematic measurements under controlled conditions, while maximally mimicking the microbiome–host interface (Jalili‐Firoozinezhad *et al*, 2019). Such *in vitro* approaches simulating microbiome–host interactions can in the future propel our mechanistic understanding of drug–microbiome–host interactions.

Altogether, *in vitro* screening approaches adapted from pharmaceutical research and novel technology‐driven platforms can both facilitate the systematic study of drug‐microbiome–host interactions. As shown for GPCRs, such approaches can provide functional insights into the molecular interactions between microbes and the host, caused by drug administration. A systematic dissection of the underlying interactions will pave the way for future *in vivo* studies in animal models and clinical settings.

### Animal models

Ultimately, we aim at understanding the effects of drug intake on the whole organism. To this end, invertebrate models can represent powerful models bridging cell culture systems to complex model organisms and cohort studies. Both the fruit fly *Drosophila melanogaster* and nematode worm *C*. *elegans*, two model organisms with well‐established genetic and genomic resources, are often overlooked in microbiome research (Norvaisas & Cabreiro, 2018). This originates from the fact that their associated microbes poorly reflect the taxonomic and functional diversity of the human microbiome and that in the case of *C. elegans*, the impact of microbes on the host is rather of nutritional than of symbiotic nature (Trinder *et al*, 2017; Douglas, 2019; Zimmermann *et al*, 2020). Both organisms have long‐standing traditions of high‐throughput screening and permit a variety of different biomedical readouts ranging from fluorescence reporters of gene function to lifespan, fertility, and behavioral investigations. Furthermore, they both present valuable advantages for microbiome studies, such as effortless large‐scale production of germ‐free or gnotobiotic animals and facile genetic manipulation, allowing for scalable, cost‐ and time‐efficient studies of the host–drug–microbiome interface (Diot *et al*, 2018; Douglas, 2018). These two invertebrate models have proven their worth for drug discovery research (Pandey & Nichols, 2011; O’Reilly *et al*, 2014; Fernández‐Hernández *et al*, 2016) and have lately been successfully employed to study interactions at the drug–microbiome–host triad.

The low diversity gut microbiome of *Drosophila melanogaster* has recently been advantageous in revealing general principles of antibiotic tolerance that are mediated by metabolic interspecies interactions (Aranda‐Díaz *et al*, 2020). In a series of elegant studies, the *C. elegans* model allowed to identify bacterial nucleotide metabolism genes that affect chemotherapeutic efficacy on the host (Scott *et al*, 2017; García‐González *et al*, 2017) or to understand how diet can affect metformin’s positive effect on lifespan by gut microbes (Pryor *et al*, 2019). In summary, invertebrate models can be instrumental in pre‐selecting the most relevant of the many possible drug–microbe combinations for a given question.

In contrast to invertebrate models, rodent models have been the standard for pharmaceutical and microbiome research for decades (Nguyen *et al*, 2015). They are suited for pharmacokinetic studies, allow using established disease models and are more relevant to human host physiology and microbiota bio‐geography. In the microbiome field, rodent models are valued for the controlled experimental manipulation of host (knockouts), microbiome (gnotobiology), and environment (*e.g*., diet) and their genetic, anatomical, and physiological relatedness to humans. These are ideal starting points to address questions on drug–microbiome–host interactions. Historically, microbiome‐mediated drug metabolism was first discovered in rats: while the anti‐inflammatory drug, salicylazosulfapyridine was metabolized in conventional animals, the parent compound remained unchanged in aseptic (antibiotic treated) rats (Peppercorn & Goldman, 1972). This was the starting point for analogous studies with other drugs under the assumption of comparable metabolic functionalities between rodent‐ and human‐associated microbes. Likewise, many decades later, the combination of genetically engineered gut commensals and gnotobiotic mice provided a system to quantitatively separate host and microbiome contribution to shared drug metabolism and assess the role of a single microbial enzyme in this interaction (Zimmermann *et al*, 2019a). Other researchers employed combinatorial therapies, i.e., antibiotics combined with the drug under investigation to unravel the influence of the microbiome on the drug’s pharmacokinetic parameters (Malfatti *et al*, 2020). Furthermore, rodent models are helpful to investigate possible therapeutic strategies to mitigate microbiome‐induced drug toxicity, such as inhibitors of the bacterial beta‐glucuronidase enzymes (Wallace *et al*, 2010; Bhatt *et al*, 2020).

There are numerous rodent studies on drug‐mediated compositional microbiome changes and their consequences on host physiology. A number have examined the short‐ and long‐term effects of antibiotics (*e.g*., Cox *et al*, 2014; Cho *et al*, 2012; Nobel *et al*, 2015; Ruiz *et al*, 2017). Increasingly, such studies also investigate the effects of non‐antibiotic drugs and diet on drug susceptibility and recovery (Ng *et al*, 2019; Cabral *et al*, 2019; Garland *et al*, 2020). While humanized mice (colonized with human microbiota) have become a cornerstone model to demonstrate causality between altered microbiome composition and host phenotype in various diseases, this strategy has so far found little use to assess whether a drug’s therapeutic effect is mediated through the microbiome. One exception is again the antidiabetic drug metformin, where fecal transplantation of metformin‐treated patients into germ‐free mice was shown to be sufficient to improve glucose tolerance of recipient mice (Wu *et al*, 2017). This approach provides a powerful tool to investigate signaling along the drug–microbiome–host axis with many conceivable ways for improvement (*e.g*., enrichment and purification steps, defined microbial consortia, *ex vivo* incubation of drugs and microbes) (Walter *et al*, 2020). Rodent models have further contributed to our understanding of how the gut microbiome impacts anticancer immunotherapy by PD‐1 (Tanoue *et al*, 2019), CTLA‐4 blockage (Vétizou *et al*, 2015; Sivan *et al*, 2015; Mager *et al*, 2020) or in cyclophosphamide therapy (Viaud *et al*, 2013), all resulting in findings of high transferability to humans (reviewed in (Zitvogel *et al*, 2018).

Comparative systems‐level analyses of gnotobiotic and conventionally raised mice make it possible to map the effects of microbial colonization at the organismal scale (Mills *et al*, 2020). Such approaches have revealed that numerous host xenobiotic processing genes, *i.e*., P450 cytochromes (CYPs), phase II enzymes and transporters are influenced by the microbiome, both at the RNA and protein level and at various body sites (Selwyn *et al*, 2016; Kuno *et al*, 2016, 2019; Fu *et al*, 2017). Hence, the microbiome can also have an indirect impact on drug pharmacokinetics by modulating xenobiotic metabolism of the host (Dempsey & Cui, 2019).

Well‐designed approaches that allow parallelizing the performed analyses and thus reducing the amount of experimental animals will tremendously accelerate our understanding of drug–microbiome–host interactions in both directions, namely those of drugs on microbes as well as those of microbes on drugs.

### Translation to human

A better mechanistic understanding of the drug–microbiome–host interactions opens the translational possibility to harness the microbiome and its interpersonal variability in composition to improve drug treatments in both general and personalized manners. Such microbiome‐based treatments could encompass a wide range of different applications (Fig 3). Analogous to human genetic markers guiding drug dosing and potential drug‐drug interaction risks, microbiome biomarkers could be used to predict drug response and guide treatment regimens, as showcased for digoxin (Haiser *et al*, 2013). The identification of microbiome‐encoded enzymes that negatively impact drug response is the basis for the development of specific inhibitors targeting these microbial processes. Such inhibitors have been developed to inhibit microbial metabolism of L‐dopa and deglucuronidation of drug metabolites (Wallace *et al*, 2010; Maini Rekdal *et al*, 2019). Although conceptually interesting, adding additional bioactive compounds to a given drug formulation comes with new challenges, such as regulatory hurdles, increased polypharmacy, and target delivery to the microbiome. Furthermore, targeting microbial enzymes bears the inherent risk of altering microbiome composition and potentially function. However, this risk also presents an opportunity. In contrast to the human genomes, the gut microbiome can be rapidly modified, uniquely allowing both sides of the patient‐drug interaction to be optimized for maximum therapeutic benefit (Taylor *et al*, 2019). Interventions such as dietary changes, antibiotic administration, or fecal microbiota transplantation (FMT), induce general shifts in microbiome composition; whereas prebiotics, probiotics and phage therapies have the potential of introducing targeted changes to the microbiome (Box 2). Aside from these interventions which aim at altering the microbiome composition more permanently, approaches to temporarily change the functional output of the microbiome have also been envisioned. Such transient changes could be achieved through the administration of probiotics that do not stably colonize the gut, but that change gut physiology during their intestinal passage. Another promising avenue is the use of postbiotics, which are the functional output of beneficial microbes, such as metabolites, that are administered abiotically.

Box 2 **Microbiome modulations**

The microbiome has become a primary therapeutic target, with many ongoing clinical trials for multiple medical indications. These studies typically aim at modulating the microbiome toward a health‐promoting state for its human host (*e.g*., for colorectal cancer (Fong *et al*, 2020), for atherosclerosis (Chen *et al*, 2020)). The means to do so vary immensely and include a range of interventions that can be separated in biotic and abiotic agents leading to either global or targeted changes of the microbiome composition. Furthermore, some of these microbiome‐targeted therapies aim at permanently altering the microbiome, whereas others aim at a transient effect. All of these interventions have in common that they alter the functional output of the microbial community and hence the microbiome–host interactions. Although, microbiome modulations have not yet been extensively explored to alter microbiome–drug–host interactions to improve drug response and alleviate adverse effects, we provide an overview of the potential means to do so (see also Fig 3).

*Abiotic interventions* consist of dietary changes that shift microbiome composition and prebiotics, which are specific compounds, such as certain sugars, that are preferred by microbiome subpopulations leading to their increase in abundance. Additional abiotic agents include peptides, drugs, and other xenobiotics, of which antibiotics are intuitive microbiome modifiers. More recently, postbiotics have gained increasing attention (Wegh *et al*, 2019). The term summarizes a variety of different bioactive fermentation products such as short‐chain fatty acids or secondary bile acids. In contrast to the other agents, postbiotics do not act via compositional microbiome changes but directly mimic an altered functional microbiome output.

*Biotic interventions* are based on biological agents, such as entire gut communities or specific microbes to modify the function of a person’s microbiota. Fecal microbiota transplantations (FMT) transfer the entire microbial gut community from one person to another. Due to the challenges to standardize and regulate fecal material (Giles *et al*, 2019), many efforts aim at engineering synthetic communities of defined quality and properties that can be transplanted. Probiotics describe specific bacterial strains intended for therapeutic purposes (Suez *et al*, 2019). They are GRAS‐certified (generally regarded as safe) by the Food and Drug Administration and include microbes from different phyla such as *Lactobacillus reuteri* and *Bifidobacterium casei*. Further, probiotic bacteria can be genetically modified to express specific therapeutic properties (*e.g*., therapeutic proteins (Gurbatri *et al*, 2020)), whereas next‐generation probiotics are based on microbial isolates from the gut microbiota that are re‐inserted, possibly after *in vitro* modification (O’Toole *et al*, 2017). The discovery of increasing numbers of microbiome‐associated bacteriophages opens the opportunity to apply phage‐therapy to eliminate unwanted bacterial species and strains from a microbial community in a very specific manner (Sausset *et al*, 2020). In preclinical models of colorectal cancer, phages of the tumor‐associated *Fusobacterium nucleatum* have even been used for targeted drug delivery (Zheng *et al*, 2019).

Microbiome‐targeting interventions could also be employed to counteract the compositional shifts introduced by medicinal drug treatments. Autologous fecal microbiota transplantation (auto‐FMT) has improved treatment outcome of patients undergoing allogeneic hematopoietic stem cell transplantation to reconstitute their microbiome after the antibiotic regimens during hematopoietic stem cell replacement (Taur *et al*, 2018). Similar approaches could be applied to treat dysbiosis induced by human‐targeted drug treatments. Two recent studies co‐administered small molecule drugs with antibiotics to alleviate the effects of antibiotics on gut commensals (preprint: Maier *et al*, 2020; Garland *et al*, 2020), providing new ways to avoid or revert the collateral damage of drug treatment on healthy microbiomes.

Apart from direct drug–microbiome interactions, indirect effects can also influence microbiome and host functions and interactions. For example, proton‐pump inhibitors (PPI)s are among the drugs with the most pronounced effect on the gut microbiome composition (Imhann *et al*, 2016). It is thought that drug‐induced changes in intestinal physiology, such as increased gastric pH, contribute to this effect. Analogously, drugs that alter intestinal motility have also been shown to impact microbiome composition (Vich Vila *et al*, 2020). The microbiome can also indirectly influence human drug metabolism and impact pharmacokinetics through regulation or inhibition of human enzymes. For example, the microbiome‐produced metabolite p‐cresol has been shown to competitively inhibit O‐sulfonation enzymes in the liver, which interferes with acetaminophen clearance (Clayton *et al*, 2009). Given the complexity of these indirect effects and our current lack of their understanding, systematic approaches are needed to comprehensively map these interactions at the molecular level.

When translating laboratory findings, in particular from high‐throughput *in vitro* screens, to human studies, it is essential to take pharmacokinetic principles into account. For example, oral drugs, which are still the major form of formulations used, are typically absorbed in the small intestine where bacterial densities are rather low. In contrast, the large intestine harbors the most dense and diverse microbial communities of the human body, but the absorption capacity of the colon is limited due to reduced surface area and number of transport proteins. This raises the question of which bacteria–drug interactions are actually relevant to drug pharmacokinetics.

To overcome these limitations, physiology‐based pharmacokinetic (PBPK) models that take into account the microbiome–drug interactions for pharmacokinetics have been recently developed (Zimmermann *et al*, 2019a; Zimmermann‐Kogadeeva *et al*, 2020). Limited bioavailability, altered intestinal transit and slow release drug formulations directly influence drug concentrations in the large intestine, which can affect reciprocal effects between the drug and the microbiome. In order to directly impact drug pharmacokinetics, microbial metabolism and/or bioaccumulation has to occur in the intestinal tube, where absorption of drug or its metabolites also takes place. An example of such direct competition between microbial drug metabolism and intestinal absorption was shown for L‐dopa in the small intestine, which reduces the absorbed drug in a microbiome‐dependent manner (Maini Rekdal *et al*, 2019). Another example is the microbial conversion of sorivudine and brivudine to the liver‐toxic metabolite bromovinyluracil in the large intestine, which leads to increased serum levels and liver toxicity following large intestinal absorption (Zimmermann *et al*, 2019a). The microbiome‐specific PBPK model further predicts that extensive biliary excretion of drug and drug metabolites can lead to their accumulation in the intestine and hence result in enhanced drug–microbiome interactions. This provides a route and explanation for non‐orally administered drugs to interact with the gut microbiome, as shown for the intravenously administered anticancer drug irinotecan (CPT‐11), for which microbiome‐caused adverse effects are dose and treatment limiting (Wallace *et al*, 2010). CPT‐11 is a prodrug that gets converted to its active compound SN‐38 by human enzymes, before phase II liver reactions (*i.e*., glucuronidation) inactivate the compound before biliary secretion into the intestine. The human gut microbiome encodes for hundreds of different beta‐glucuronidases that remove glucuronate from liver‐derived (drug) metabolites (Pollet *et al*, 2017). In the case of SN38, this leads to local accumulation of the cytotoxic compound resulting in intestinal complications, such as severe diarrhea. In other cases, microbiome‐produced deglucuronidation products can be either directly or following additional microbial conversion re‐absorbed from the intestine impacting systemic drug or drug metabolite exposure, as shown for mycophenolate and clonazepam, respectively (Elmer & Remmel, 1984; Ishizaki *et al*, 2012; Zimmermann *et al*, 2019a). Although, systematic approaches are needed to comprehensively map possible drug–microbiome–host interactions at the molecular level, these examples emphasize the importance of keeping an organismal view on the results and evaluating their relevance for translatability to clinics.

## Conclusions and future perspectives

Systematic approaches have started to provide new insights into drug–microbiome–host interactions. A molecular understanding of the drug–microbiome–host triad will open up strategies to fine‐tune existing clinical drug treatments, improving drug efficacy and reducing adverse effects. Such strategies could encompass both microbiome‐inspired compound modifications and modulations of the microbiome composition. It is conceivable that approaches geared toward a systematic assessment of drug–microbiome interactions will become part of preclinical drug development, complementing current *in vitro* assays and *in silico* modeling to predict human drug metabolism and toxicity. In addition to the need of taking into account drug–microbiome–host interactions for new regulatory requirements, such knowledge can be key at preclinical drug development stages. It can improve early compound triaging and target studies on specific patient groups, foreseeing complications that may arise in the clinical phase due to interpersonal microbiome differences.

An improved mechanistic understanding of reciprocal drug–microbiome interactions will also shed light on the potential role of the microbiome in drug–drug interactions, often observed in polypharmacy. More broadly, non‐drug compounds, such as other xenobiotics, nutrients, excipients, and even endogenous metabolites, likely influence drug–microbiome–host interactions as well. Therefore, we first need to systematically map these interactions and then to understand their mechanistic base to empower future (personalized) treatments. Big part of the future of drug discovery lies in harnessing the patients’ microbiome composition and function for an improved therapeutic outcome.

## Conflict of interest

The authors declare that they have no conflict of interest.
